# Multicargo Porous Cochlear Electrode Coating for Antifibrosis After Cochlear Implantation

**DOI:** 10.1002/advs.202412158

**Published:** 2025-05-12

**Authors:** Lei Ren, Yangnan Hu, Xiaoqiong Ding, Menghui Liao, Tian Shen, Sixing Cao, Hui Zhang, Hong Cheng, Yanru Qi, Pan Feng, Xinyi Pang, Ling Lu, Huan Wang, Wenwen Liu, Renjie Chai, Lin Cheng

**Affiliations:** ^1^ State Key Laboratory of Bioelectronics Department of Otolaryngology Head and Neck Surgery Zhongda Hospital School of Life Sciences and Technology Advanced Institute for Life and Health Jiangsu Province High Tech Key Laboratory for Bio‐Medical Research Southeast University Nanjing 210096 China; ^2^ Co‐Innovation Center of Neuroregeneration Nantong University Nantong 226001 China; ^3^ Department of Otolaryngology‐Head & Neck Surgery West China Hospital Sichuan University Chengdu 610041 China; ^4^ College of Food Science and Engineering Nanjing University of Finance and Economics Nanjing 210023 China; ^5^ The Eighth Affiliated Hospital Sun Yat‐Sen University Shenzhen 518033 China; ^6^ Department of Otolaryngology‐Head and Neck Surgery Shandong Provincial ENT Hospital Shandong University Jinan 250022 China; ^7^ Department of Neurology Aerospace Center Hospital School of Life Science Beijing Institute of Technology Beijing 100081 China; ^8^ Department of Otolaryngology Head and Neck Surgery Sichuan Provincial People's Hospital University of Electronic Science and Technology of China Chengdu 610000 China; ^9^ Southeast University Shenzhen Research Institute Shenzhen 518063 China

**Keywords:** antifibrosis, cochlear electrode, hydrogel, porous PDMS, tissue engineering

## Abstract

Severe to profound sensorineural hearing loss seriously affects the communication and cognitive ability of the patients. Cochlear implantation (CI) is currently the most effective treatment, while it may damage the remaining inner ear function due to its poor biocompatibility and the resultant fibrosis. Herein, a porous methacrylated poly(dimethylsiloxane) (MA‐PDMS)‐coated cochlear electrode is presented for CI and hearing protection. The porous MA‐PDMS is filled with a hybrid hydrogel system made of dexamethasone sodium phosphate (Dex), Ti_3_C_2_T_x_ MXene (MXene), and methacrylate gelatin (GelMA). The coating shows good biocompatibility and drug loading and release capacity in vitro, protective effects on hair cells (HCs) and spiral ganglion neurons (SGNs) of the inner ear, as well as the residual hearing protection and the effective fibrosis reduction in vivo. It is anticipated that this porous electrode drug‐loading coating may provide a valuable reference strategy for the future cochlear electrode transplantation system.

## Graphical abstract

This study presents a novel porous drug‐loading coating for cochlear electrodes, utilizing MA‐PDMS infused with GelMA hydrogel containing dexamethasone and MXene nanoparticles. The coating enhances biocompatibility, enables sustained drug release, reduces fibrosis, and preserves residual hearing post‐CI. This approach offers a promising strategy for improving cochlear electrode design and clinical outcomes.

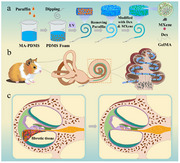

## 1 Introduction

The World Health Organization World Hearing Report 2021 highlights that 20% of the global population suffers from hearing impairment. Sensorineural hearing loss (SNHL) accounts for the major portion and is mainly caused by permanent injury to hair cells (HCs) or spiral ganglion neurons (SGNs).^[^ ^1^, ^2^, ^3^ ^]^ Cochlear implants (CIs), which converts acoustic signals into electrical signals and directly stimulates the SGNs to replace damaged cochlea, is currently considered as the most effective treatment for severe to profound SNHL.^[^ ^4^, ^5^, ^6^ ^]^ However, an inflammatory foreign body reaction caused by CI electrode insertion is unavoidable. In addition, fibrous sheath can form around the implanted electrodes, disrupting the normal structure of the inner ear and leading to apoptosis of residual HCs and SGNs.^[^ ^7^, ^8^ ^]^ As a result, the physiology and mechanics of the cochlea, as well as the impedance and conductivity of the electrodes, can be affected, even leading to complete loss of residual hearing.^[^ ^9^, ^10^, ^11^ ^]^ Therefore, novel improvements to cochlear electrodes are expected.

In this work, we propose a porous hydrogel hybrid drug‐loading coating for electrode encapsulation to achieve an improvement of CI electrodes. Porous poly(dimethylsiloxane) (PDMS) is an elastomer with unique properties such as good biocompatibility, great resistance to biodegradation ability, and excellent mechanical properties,^[^ ^12^ ^]^ which make it a promising material for encapsulation implantable biomedical microdevices.^[^ ^13^ ^]^ Moreover, the porous structure endows it with a high specific surface area and adjustable biological functions,^[^ ^14^ ^]^ which enables it to load active molecules and is considered as a promising candidate for drug delivery.^[^ ^15^, ^16^, ^17^ ^]^ However, this technique of encapsulation through porous PDMS to improve the performance of implants has not yet been used on CI.

Herein, gelatin methacrylate (GelMA) encapsulating dexamethasone sodium phosphate (Dex) and Ti_3_C_2_Tx MXene (MXene) was infused into the pores of methacrylated PDMS (MA‐PDMS) and polymerized to prepare a porous electrode drug‐loading coating. GelMA is an extracellular matrix‐like hydrogel with adjustable mechanical properties, excellent photo‐crosslinking properties, and good biocompatibility.^[^ ^18^ ^]^ The combination of GelMA hydrogels with bioactive molecules and functional nanomaterials can form a multifunctional platform for tissue therapeutics.^[^ ^19^, ^20^, ^21^ ^]^ MXene is a two‐dimensional material with an ultrathin layered structural topology, high specific surface area, good biocompatibility, and excellent photothermal conversion ability. As a non‐enzymatic nano‐antioxidant, MXene can alleviate the damage caused by oxidative stress.^[^ ^22^ ^]^ Clinically, glucocorticoids are often used locally or systemically to inhibit cochlear fibrosis and protect the residual hearing after CI.^[^ ^23^, ^24^ ^]^ Studies have demonstrated that the electrode surface coating remains unaffected by electrical stimulation and can achieve long‐term implantation effects.^[^ ^25^, ^26^ ^]^ More notably, electrode coatings containing dexamethasone can prevent an increase in electrode impedance without adversely affecting the electrochemical properties of the electrodes.^[^ ^26^, ^27^ ^]^ Based on these advantages, we demonstrated that the GelMA‐MXene‐Dex hydrogel could preserve SGNs, protect the residual hearing, especially the low‐frequency hearing, and reduce the fibrosis after CI in vivo. Distinct from conventional hydrogel coatings that are merely applied to electrode surfaces or electrode arrays,^[^ ^25^, ^26^, ^27^, ^28^, ^29^, ^30^, ^31^, ^32^, ^33^, ^34^, ^35^, ^36^, ^37^, ^38^ ^]^ our methodology involves the integration of a therapeutic agent‐containing nanocomposite hydrogel within the porous architecture of the electrode. This innovative design demonstrates enhanced structural stability over traditional hydrogel coatings, while facilitating controlled release of both nanoparticles and pharmacological agents without compromising electrode array functionality. These features indicated that the multicargo porous electrode drug‐loading coating may provide a new strategy for novel CI electrode coating.

## 2 Results and Discussion

**Figure 1:**
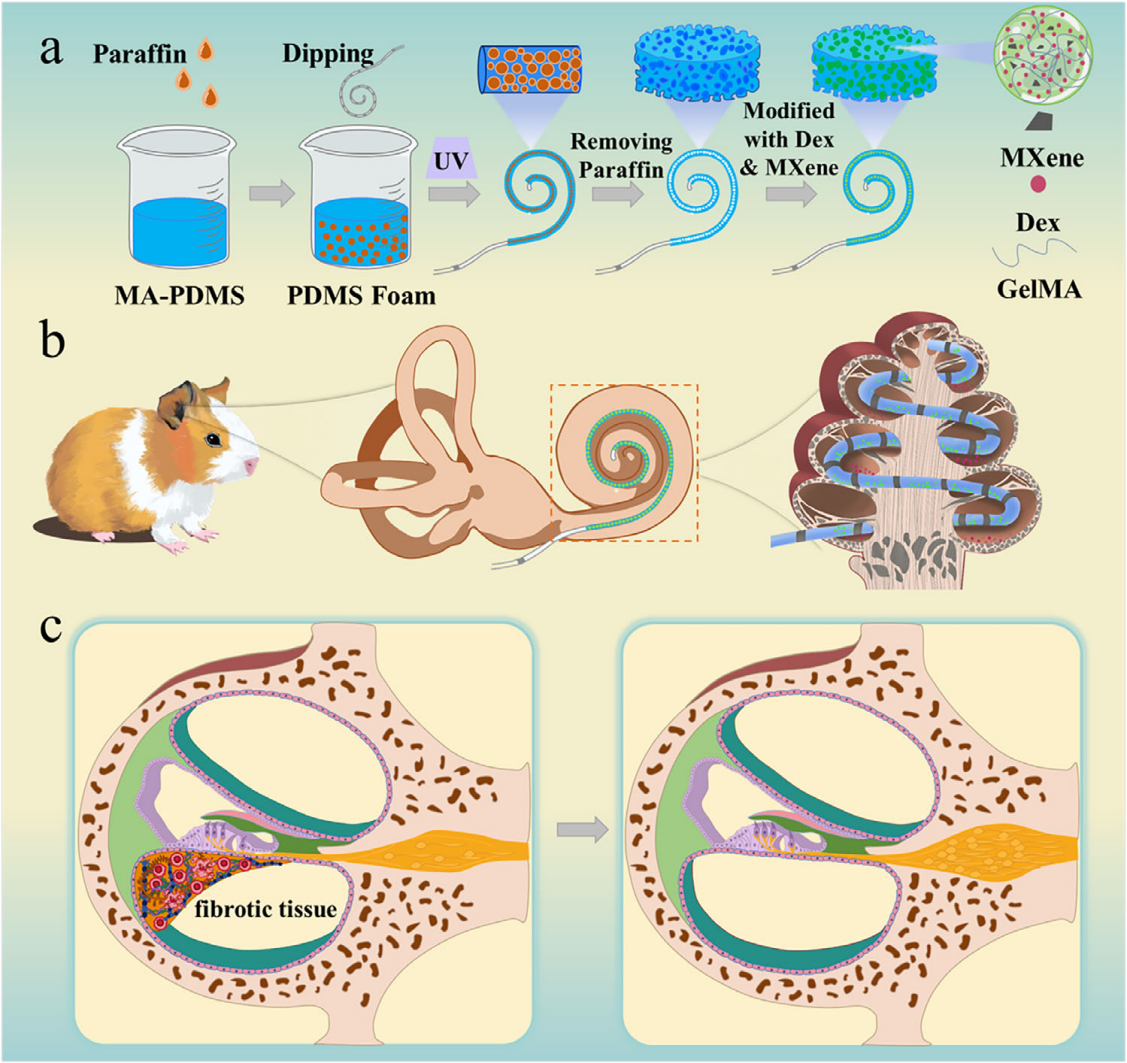
Schematics of multicargo porous electrode drug‐loading coating for antifibrosis after cochlear implantation (CI). a) The synthesis of multicargo porous electrode drug‐loading coating. b) The schematic diagram of CI. c) The scheme of the performance of repairing the Corti organ damage, preserving SGNs, and preventing fibrosis in scala tympani (ST) after multicargo porous electrode insertion.

**Figure 2:**
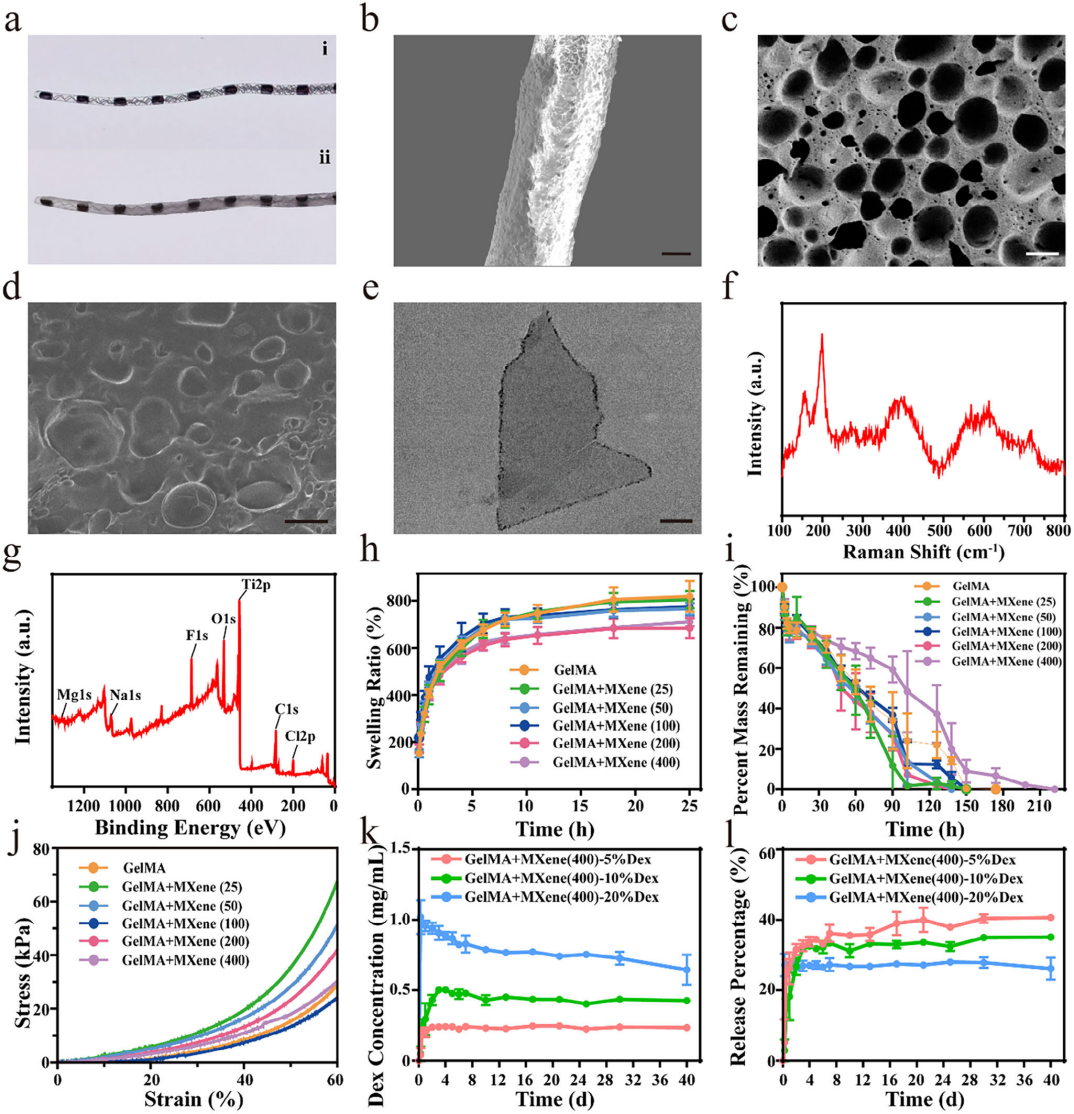
Characterization of the CI electrodes and GelMA‐MXene hydrogels. a) Digital photographs of the conventional CI electrode (i) and coated porous drug‐loading cochlear electrode (ii). b,c) SEM images of the MA‐PDMS porous structure coated on the surface of CI electrode at low‐(b) and high‐magnification (c), scale bar indicates 200 µm in (b) and 20 µm in (c). d) SEM image of the GelMA‐MXene hydrogel filled MA‐PDMS porous structure, scale bar indicates 100 µm. e) High‐resolution TEM image of an MXene nanosheet, scale bar represents 200 nm. f) Raman spectrum of MXene. g) XPS pattern of MXene. h–j) The swelling ratio (h), degradable behaviors (i), and mechanical stress testing outcomes (j) for the GelMA‐MXene hydrogel with different MXene concentrations. k) The real‐time Dex concentration released from the GelMA‐MXene hydrogels for 40 days (*n* = 3). l) The release percentage of Dex from the GelMA‐MXene hydrogels for 40 days (*n* = 3). Data are presented as mean ± standard deviation (SD).

In a typical experiment, we used the emulsion template method to fabricate the porous MA‐PDMS structures, as shown in **Figure** **1**. Liquid paraffin and span 20 were employed as porogen and surfactant respectively, to prepare porous MA‐PDMS, while n‐hexane and photoinitiator were added to improve the uniformity of the solution and to endow it with the ability of ultraviolet (UV) light‐triggered polymerization. The cochlear electrode was then modified with the prepared porous MA‐PDMS and cured under UV light 3 times to obtain the coated electrode. After the electrodes were immersed in anhydrous ethanol to remove the solvent, the pores of the porous MA‐PDMS were filled using GelMA‐MXene hydrogel to obtain a porous electrode drug‐loading coating, as shown in **Figure** **2**a and Figure S1 (Supporting Information). Subsequently, the structure of porous electrode drug‐loading coating was observed by scanning electron microscopy (SEM). Figure 2b–d demonstrates the MA‐PDMS porous structure on the electrode surface and the microstructure after being filled with GelMA‐MXene hydrogel, respectively. We conducted a thorough characterization of the porous MA‐PDMS, and the results showed that its porosity is 76.70% ± 2.29%, with pore sizes of 38.65 ± 9.61 µm, as illustrated in Figure S2 (Supporting Information). Density measurements revealed that the porous MA‐PDMS exhibited a density of 0.787 ± 0.093 g cm^−^^3^, which changed to 1.094 ± 0.005 g cm^−^^3^ following GelMA hydrogel infiltration, as shown in Figure S3 (Supporting Information). Figure S4 (Supporting Information) displays the force‐displacement curves for porous MA‐PDMS compared to porous MA‐PDMS infused with GelMA hydrogels (Figure S4b, Supporting Information). Furthermore, with the infusion of GelMA hydrogel, the shear strength of the porous MA‐PDMS decreases. Porous MA‐PDMS exhibited a shear strength of 55.54 ± 10.93 kPa, which was higher than porous MA‐PDMS filled with GelMA hydrogels 26.19 ± 8.44 kPa, as shown in Figure S4c (Supporting Information). We also evaluated the hydrophilicity of the different coatings by conducting water contact angle tests. As shown in Figure S5 (Supporting Information), compared to the uncoated control group, the water contact angles for the porous MA‐PDMS filled with GelMA hydrogels and the porous MA‐PDMS filled with GelMA‐MXene hydrogels were significantly reduced. This finding suggests that our multicargo coating exhibits superior hydrophilic properties.

Afterward, we performed a series of characterisations of the MXene used, including transmission electron microscopy (TEM), Raman spectroscopy, and X‐ray photoelectron spectroscopy (XPS). MXene was synthesized through chemical etching of Ti_3_AlC_2_ using a mixture of HCl and LiF to remove Al layers. As shown in Figure 2e, the synthesized MXene had the morphology of a monolayer nanosheets with the dimensions of each nanosheet of about a few hundred nanometres Raman spectroscopy showed characteristic vibrations corresponding to surface groups attached to titanium and carbon, occurring in the spectral ranges of 230–470 cm^−1^ and 580–730 cm^−1^, respectively (Figure 2f). Besides, the full XPS spectrum confirmed that the primary composition of the prepared nanosheets are Ti, C, and O, consistent with the typical elemental profile of MXenes (Figure 2g). We further evaluated the surface ultrastructure, crosslinking degree, and mechanical properties of GelMA‐MXene hydrogels. Figure S6 (Supporting Information) showed the SEM images of GelMA‐MXene hydrogel at MXene concentration of 0, 25, 50, 100, 200, and 400 µg mL^−1^, respectively. The results indicated that the GelMA‐MXene hydrogels also exhibited a porous structure at all concentrations compared to the pure GelMA hydrogels, suggesting that the incorporation of MXene did not alter the 3D architecture of the GelMA hydrogels. Figure 2h,i displays the swelling and degradation rates of GelMA‐MXene hydrogels. The data revealed that an increase in MXene concentration corresponds to a decrease in the degree of swelling, as depicted in Figure 2h. High swelling rates can potentially lead to increased tension at the tissue‐hydrogel interface, resulting in the detachment of adhered hydrogels. Therefore, the reduced swelling proportion upon MXene incorporation theoretically enhances the stability of the prepared polymeric system. contributing favorably to maintaining the performance of hydrogel in biological environments. Subsequently, we observed the degradation rate of GelMA‐MXene hydrogels with varying MXene concentrations in a solution containing 1 U mL^−1^ collagenase. As shown in Figure 2i, it was observed that both pure GelMA and the GelMA‐MXene hydrogels showed a slow degradation rate and could completely degrade. We found that when the concentration of MXene was 200 µg mL^−1^ or less, the degradation rate of the GelMA‐MXene hydrogels were faster than that of pure GelMA; however, when the MXene concentration reached 400 µg mL^−1^, the degradation rate of the GelMA‐MXene hydrogels significantly slowed compared to pure GelMA. This biodegradable implant avoids premature collapse of the drug carrier, thus providing essential physical support for sustained drug release. Additionally, mechanical stress tests were conducted on the resulting GelMA‐MXene hydrogels. It was found that the mechanical stress levels of the GelMA‐MXene hydrogels were comparable to those of the pure GelMA hydrogels, indicating that the incorporation of MXene did not adversely affect the mechanical durability and structural integrity of the GelMA hydrogels (Figure 2j).

Currently, the clinical approaches for addressing fibrosis following CI surgery primarily involve the use of Dex. However, systemic drug administration for inner ear disorders faces limitations, particularly in overcoming the blood‐labyrinth barrier to ensure effective drug delivery to the target site. Consequently, higher doses may be required to attain therapeutic concentrations, which often entail substantial adverse reactions, thereby restricting the safety and feasibility of long‐term usage.^[^ ^39^ ^]^ To overcome this challenge, the drug was coated on the surface of porous electrodes by the GelMA hydrogels containing Dex and MXene were filled into the MA‐PDMS holes on the surface of CI electrodes. This novel electrode design aspires to improve biocompatibility between the electrode and biological tissue and enable sustained drug release functionality. GelMA‐MXene hydrogels with different concentrations of Dex (mass fractions of 5%, 10%, and 20%) were prepared to investigate the influence of drug concentration on the GelMA‐MXene hydrogels performance and drug release behavior. The release of Dex from the GelMA‐MXene hydrogels were then examined by immersing them in artificial perilymph at 37 °C, and the change in drug concentration over time was determined using UV–vis spectrophotometry. As shown in Figure 2k, the release of Dex from the hydrogels exhibited an initial burst, followed by a sustained release, maintaining a stable concentration for at least 40 days. When the mass fraction of Dex was 20%, the total release rate is the lowest (26.16% ± 3.11%), and reached the peak within 6 h (Figure 2l; Figure S7, Supporting Information). When the Dex mass fraction of Dex was reduced to 10%, the release rate of Dex within 40 days was moderate (35.10% ± 0.13%), with a sustained release profile that peaked at the Dex concentration after 3 days. Meanwhile, when the mass fraction of Dex was 5%, the total release rate was the highest (40.62% ± 0.88%), with the peak Dex concentration achieved within 12 h. Considering the balance between time and sustained release rate, we chose 10% Dex for further electrode coating preparation.

To assess the feasibility of applying coated cochlear electrodes, we first studied the in vitro cytotoxicity of GelMA‐MXene hydrogels on House Ear Institute‐Organ of Corti 1 (HEI‐OC1) cells and SGNs. After culturing with the exudate of GelMA‐MXene hydrogel for 48 h, the Live/Dead assay and Cell Counting Kit‐8 (CCK‐8) were used to evaluate its biocompatibility. It was found that the MXene concentration had no significant influence on the density of HEI‐OC1 cells and SGNs, with fewer dead cells in each group (Figure S8a,b, Supporting Information). Figure S8c (Supporting Information) reveals that the addition of MXene had no substantial effect on the viability of HEI‐OC1 cells. Conversely, when the concentration of MXene in the hydrogel was increased to 100 µg mL^−1^, the vitality of SGNs significantly improved after co‐culture, as shown in Figure S8d (Supporting Information). Considering that SGNs are non‐proliferative, we speculate that there might have been a contamination of glial cells in the extracted SGN population, and that the GelMA‐MXene hydrogel may have significantly enhanced the viability of these glial cells. In summary, the GelMA‐MXene hydrogel not only exhibits good biocompatibility, but it may also improve cell conditions and enhance cell viability.

**Figure 3:**
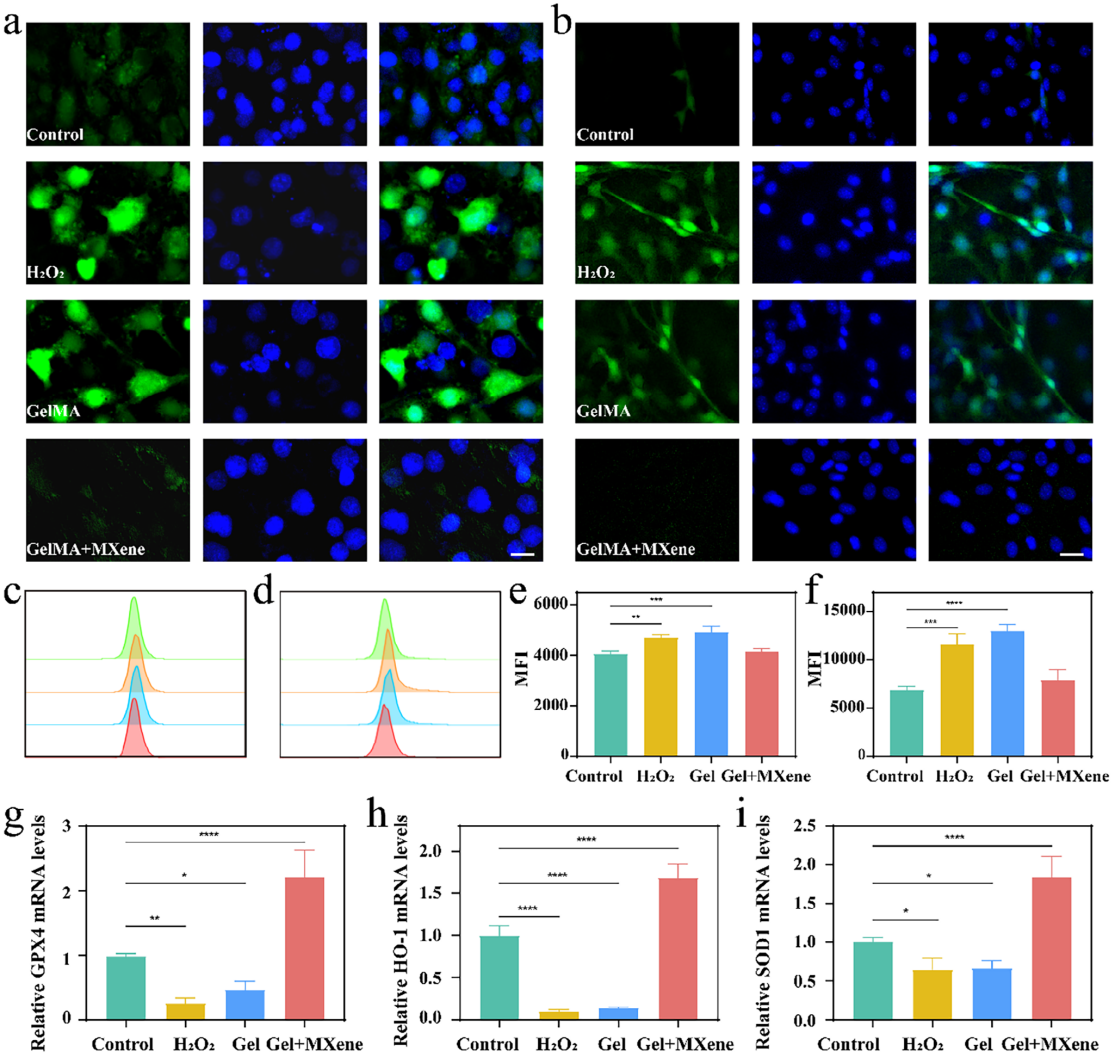
ROS generation in HEI‐OC1 cells and SGNs cultured under diverse conditions. a,b) Fluorescent micrographs of HEI‐OC1 cells (a) and SGNs (b), stained with Hoechst 33 258 for nuclei and DCFH‐DA for intracellular ROS. Scale bars indicate both 20 µm. c,d) Flow cytometric analysis outcomes for ROS levels of HEI‐OC1 cells (c) and SGNs (d) across different culture media. e,f) Quantitative flow cytometry data showing ROS levels in HEI‐OC1 cells (e) and SGNs (f) under varied media conditions (*n* = 3). g–i) GPX4 (g), HO‐1(h), and SOD1(i) mRNA expression levels in HEI‐OC1 cells under varied media conditions by quantitative real‐time polymerase chain reaction (RT‐qPCR) (*n* = 4). Data are presented as mean ± SD. *p* > 0.05; *p < 0.05; **p < 0.01; ***p < 0.001; ****p < 0.0001, one‐way ANOVA. MFI, mean fluorescence intensity; Gel, GelMA; Gel + MXene, GelMA + MXene.

Abundant research has demonstrated that reactive oxygen species (ROS) play a pivotal role in the damage of HCs and the subsequent development of SNHL.^[^ ^40^, ^41^ ^]^ Therefore, eliminating ROS is a potential strategy for cellular and tissue protection. We used the green fluorescence probe DCFH‐DA to evaluate the intracellular ROS levels in HEI‐OC1 cells and SGNs after different treatments in a hydrogen peroxide‐induced oxidative stress model (**Figure** **3**a,b). When compared to HEI‐OC1 cells and SGNs cultured in the negative control group (control group, tissue culture polystyrene, untreated with H_2_O_2_), cells cultured in the positive control group (H_2_O_2_ group, tissue culture polystyrene, treated with H_2_O_2_) and the GelMA hydrogels group (GelMA group, treated with H_2_O_2_) demonstrated a notable increase in green fluorescence intensity upon exposure to H_2_O_2_. Conversely, cells grown with GelMA‐MXene hydrogels (GelMA + MXene group, treated with H_2_O_2_) exhibited a significant decreased green fluorescence intensity, indicating the effective elimination of ROS by the GelMA‐MXene hydrogels. The results obtained from flow cytometry (as illustrated in Figure 3c,d) are in agreement with those derived from fluorescence staining using the DCFH‐DA dye. Specifically, both the H_2_O_2_ group and the GelMA group exhibited a significant increase in green fluorescence intensity when compared to the control group. Notably, when the MXene concentration reached 400 µg mL^−1^, the GelMA + MXene group demonstrated a marked reduction in green fluorescence intensity, showing no significant difference compared to the control group, as visually demonstrated in Figure 3e,f. To further study the antioxidant mechanism of GelMA‐MXene hydrogels, we conducted a thorough quantitative analysis of the mRNA levels of superoxide dismutase 1 (SOD1), heme oxygenase 1 (HO‐1), and glutathione peroxidase 4 (GPX4) in HEI‐OC1 cells across various groups. The results (shown in Figure 3g–i) revealed a significant downregulation of key antioxidant enzymes such as SOD1, HO‐1, and GPX4 in both the H_2_O_2_ and GelMA groups compared to the control group. However, in the GelMA + MXene group, the expression of these antioxidant enzymes was notably upregulated. This finding further robustly demonstrates that the nanoparticle MXene effectively maintains redox balance within cells by promoting the expression of antioxidant enzymes like SOD1, HO‐1, and GPX4, thereby significantly enhancing the antioxidant capacity of HEI‐OC1 cells and providing potent protection against damage induced by oxidative stress and ROS. In light of the GelMA‐MXene hydrogel's exceptional physicochemical properties, biocompatibility, and pronounced antioxidant characteristics, we have chosen 400 µg mL^−1^ as the preferred MXene concentration for subsequent experiments.

**Figure 4:**
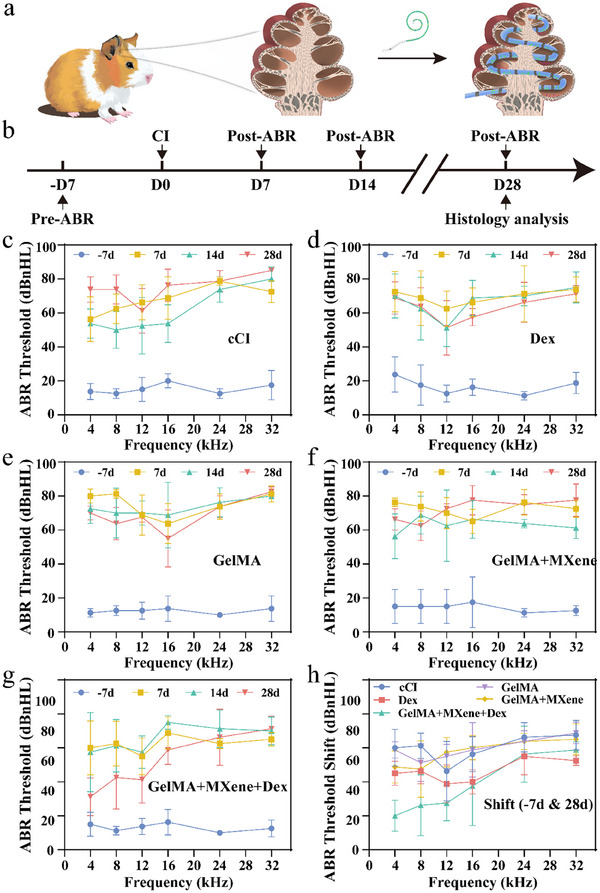
Results of animal studies and ABR. a) CI procedure in guinea pigs. b) Experiment timeline: Baseline hearing was assessed before CI. Hearing was tested at pre‐CI (−7 days) and 7, 14, and 28 days post‐CI, with histological sampling occurring on day 28. c–g) Audiometric thresholds at frequencies ranging from 4 to 32 kHz for 5 groups, measured at pre‐CI (−7 days) and 7, 14, and 28 days post‐CI (*n* = 4). h) The ABR threshold shift between pre‐CI (−7 days) and 28 days post‐CI of 5 groups (*n* = 4). Data are presented as mean ± SD.

It is assumed that the porous electrode drug‐loading coating we constructed is expected to improve the physical and chemical properties of the CI electrode, and directly release effective drugs around the perilymph, effectively preventing postoperative fibrosis and protecting residual hearing after implantation (**Figure** **4**a). In order to verify ability of this system in anti‐fibrosis and protection of residual hearing, we performed auditory brainstem responses (ABR) tests on guinea pigs at 7 days before implantation and 7, 14, and 28 days after implantation, as shown in Figure 4b. We established five groups: the group receiving unmodified conventional electrode implantation (cCI group), the group receiving unmodified conventional electrode implantation followed by Dex injection (Dex group), the group receiving porous‐modified electrode implantation with a secondary infusion of GelMA hydrogel (GelMA group), the group receiving porous‐modified electrode implantation with a secondary infusion of GelMA‐MXene hydrogel (GelMA + MXene group), and the group receiving porous‐modified electrode implantation with a secondary infusion of GelMA‐MXene‐Dex hydrogel (GelMA + MXene + Dex group). The ABR results showed that the cCI group, Dex group, GelMA group, and GelMA + MXene group had similar hearing level at 28 days after CI, with a noticeable elevation in thresholds across all frequencies compared to pre‐CI levels (Figure 4c–g). In contrast, the GelMA + MXene + Dex group exhibited the least threshold shift at 4 and 8 kHz at day 28 postoperatively (Figure 4h), indicating significant preservation of hearing at these two frequencies (Figure S9 Supporting Information). These results may be attributed to the 4 and 8 kHz regions being far from the implanted electrode, which may not be easily affected by electrode insertion. Meanwhile, 12 kHz might represent the region closest to the implanted electrode, while the 16, 24, and 32 kHz regions are directly affected by the electrode's presence. Our findings demonstrated that the proposed porous electrode drug‐loading coating effectively preserves residual hearing in guinea pigs after CI, especially at lower frequencies, which is crucial for CI procedures and holds significant clinical significance.^[^ ^42^ ^]^

**Figure 5:**
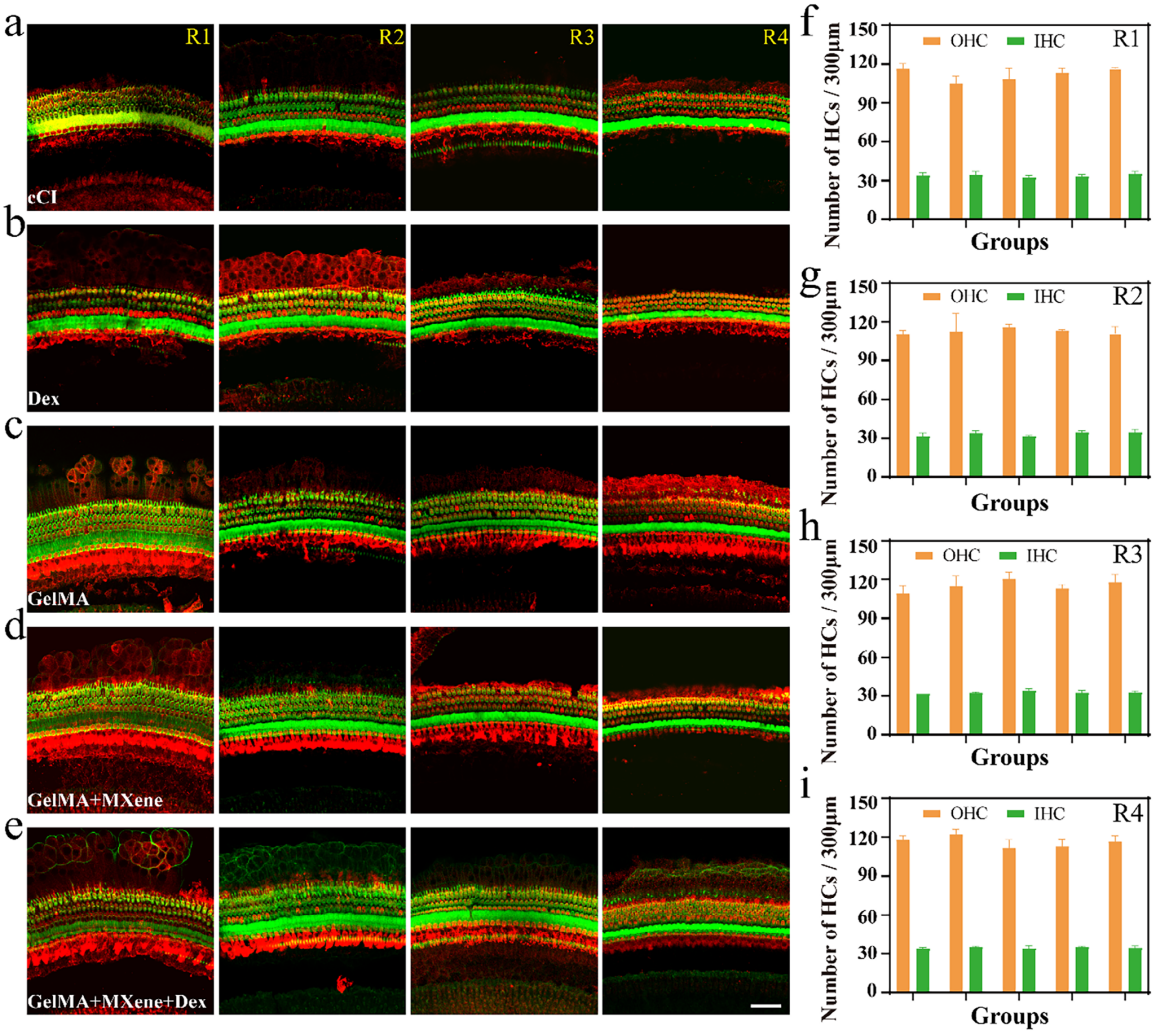
Impacts of implant treatments on hair cells (HCs). a–e) Illustrative immunofluorescence images featuring myosin 7a (red) and phalloidin (green) as HC indicators, taken from the apical (R1), upper middle (R2), lower middle (R3), and basal (R4) cochlear regions, 28 days post‐CI in five experimental groups. Scale bar = 50 µm. f–i) The survival percentage of OHCs and IHCs at the R1 (f), R2 (g), R3 (h), and R4 (i) turns from the basilar membrane of the cochlea 28 days after CI (*n* = 3). From left to right, the groups are the cCI, the Dex, the GelMA, the GelMA + MXene, and the GelMA + MXene + Dex, respectively. Data are presented as mean ± SD. *p* > 0.05; *p < 0.05, one‐way ANOVA.

Subsequently, 28 days after guinea pigs underwent CI, we conducted immunofluorescence staining with myosin 7a and phalloidin on their cochlear basilar membranes, aiming to delve deeper into the specific impact of the surgery on cochlear HCs. As illustrated in **Figures** **5**a–e, these images meticulously depict the distribution of HCs across the entire length of the cochlear basilar membranes in 5 groups of guinea pigs, spanning from the apical turn, through the upper‐middle and lower‐middle turns, to the basal turn. Reassuringly, upon close examination, no apparent signs of HC damage were observed along the entire basilar membrane. To further validate this observation, we subsequently conducted a detailed count of inner and outer hair cells (IHCs and OHCs) in specific regions of 5 groups of guinea pigs. Statistical data revealed no significant difference in the number of IHCs and OHCs among the groups (see Figure 5f–i for details). This result robustly demonstrates that cochlear implantation surgery does not exert a significant negative impact on sensorineural elements on the basilar membrane, which aligns well with the conclusions drawn from a previous research report^[^ ^34^ ^]^ (shown in Table S1, Supporting Information). Notably, the insignificant loss of HCs across all groups cannot be attributed to the protective effect of drug delivery on these structures. These research findings not only reaffirm the exceptional biocompatibility exhibited by cochlear implant electrodes but also underscore the exceptional skill and precision of surgeons during the implantation procedure.

**Figure 6:**
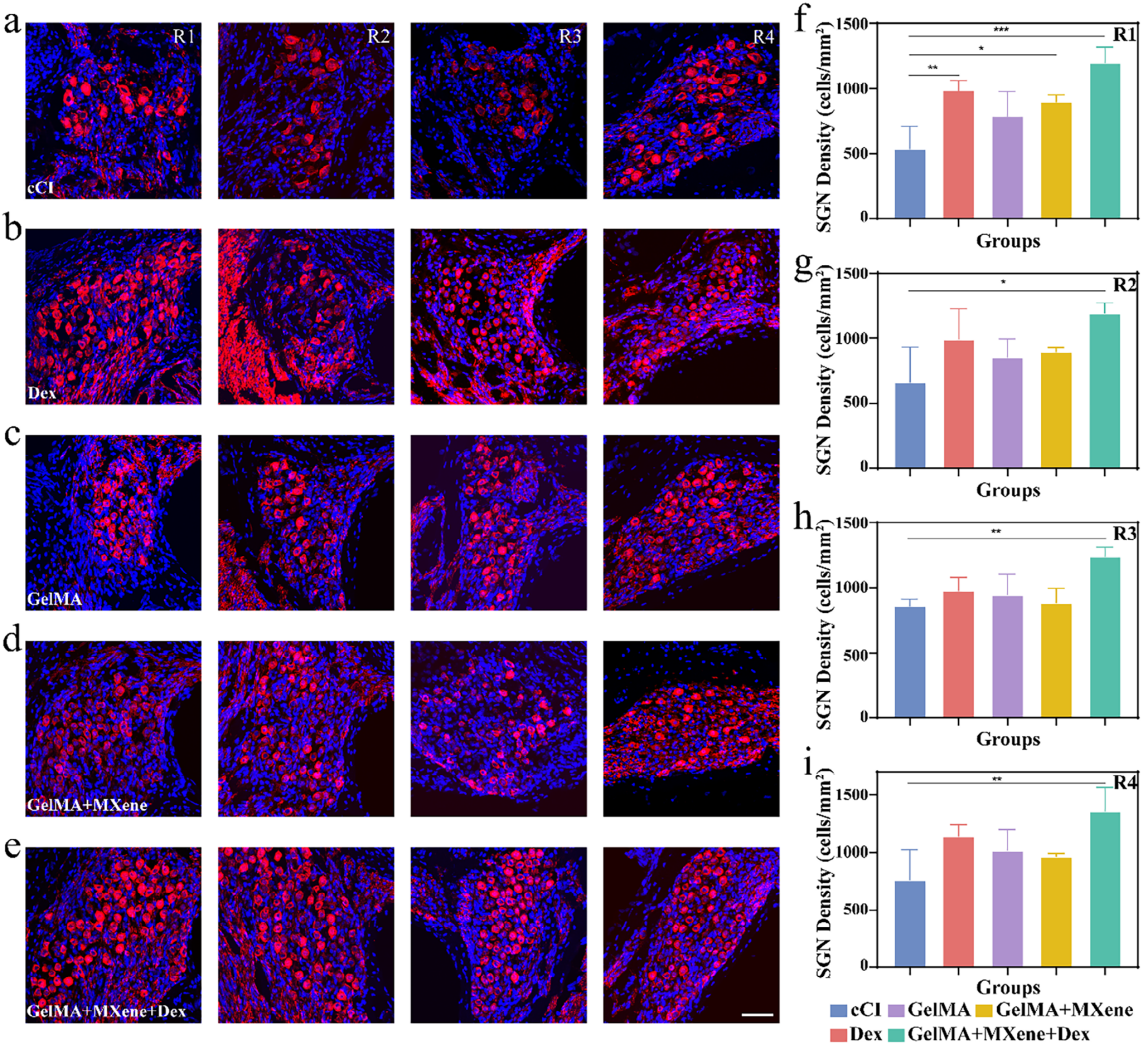
Impact of various implant treatments on SGNs. a–e) Illustrative immunofluorescence images showing Tuj‐1 (in red) and DAPI (in blue) marking SGNs at turns R1 to R4, 28 days post‐implantation across five groups. Scale: 50 µm. f–i) Quantification of SGN density at R1 to R4t urns, 28 days after CI in the five groups (*n* = 3). Groups are the cCI, the Dex, the GelMA, the GelMA + MXene, and the GelMA + MXene + Dex, respectively. Data are presented as mean ± SD. *p* > 0.05; *p < 0.05; **p < 0.01; ***p < 0.001, one‐way ANOVA.

To further assess the protective effect of multicargo porous electrode drug‐loading coating, the progressive damage to SGNs in the apical, upper middle, lower middle, and basal turn was studied. The results revealed that in the cCI group, there is a substantial loss of SGNs compared with other groups, and the damage becomes increasingly severe from the base to the apex (**Figure** **6**a–e). In contrast, the Dex group, the GelMA + MXene group, and the GelMA + MXene + Dex group exhibited significant increases in SGN density in the apical turn (Figure 6f). Nevertheless, only the GelMA + MXene + Dex group had a significant increase in the basal, lower middle, and upper middle turns compared to the cCI group (Figure 6g–i). These results suggested that Dex exhibited a favorable protective effect on SGNs and the multicargo porous electrode drug‐loading coating improved the biocompatibility of CIs while serving as an active molecular carrier, effectively preserving the residual hearing after CI implantation by promoting SGN survival. Notably, the GelMA + MXene + Dex group demonstrated the most favorable therapeutic effects due to the synergistic effects of the drug and hydrogel, with significantly elevated SGN densities observed across all turns.

**Figure 7:**
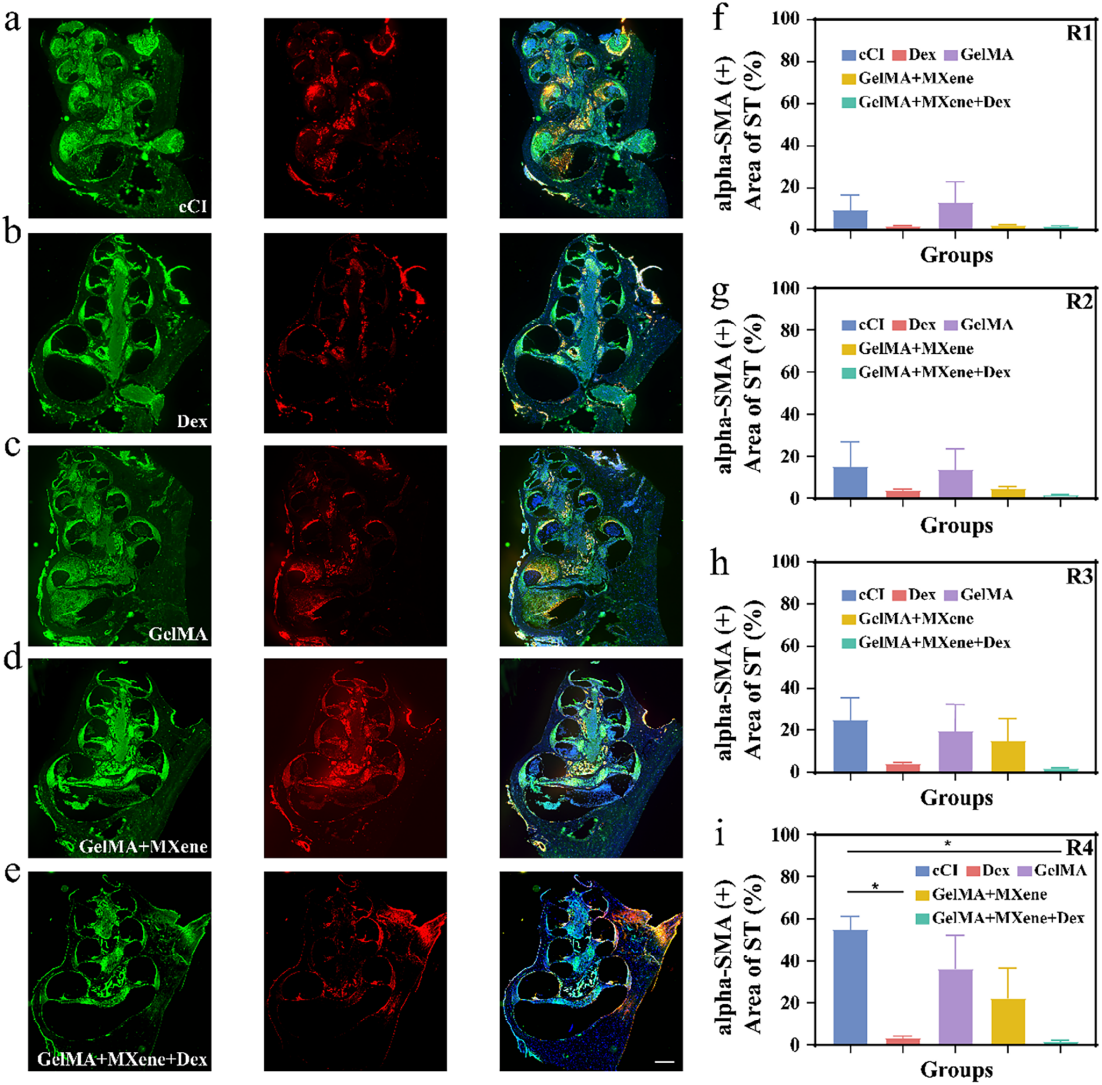
Typical confocal images of fibrosis expressed in all turns of the cochlea. a–e) Representative immunofluorescence staining for alpha‐SMA (red), Collagen I (green), and DAPI (blue) as markers for fibrosis on the ST at R1‐R4 turns from cross sections of the cochlea 28 days after CI of 5 groups. Scale bar = 500 µm. f–i) The ratio of fibrotic tissue area of ST at the R1 (f), R2 (g), R3 (h), and R4 (i) turn from cross sections of the cochlea 28 days after CI of 5 groups (*n* = 3). Data are presented as mean ± standard error of the mean. *p* > 0.05; *p < 0.05, one‐way ANOVA.

The histopathological study of patients with CI revealed foreign body tissue reactions in the cochlea, such as the fibrotic tissue and new bone formation.^[^ ^43^, ^44^ ^]^ Post‐implant fibrosis has also been reported in animal studies as well.^[^ ^45^, ^46^ ^]^ Foreign body tissue reactions following CI may cause serious consequences, including the loss of sensorineural elements, delayed low‐frequency residual hearing loss, increased electrode impedance, limited speech‐hearing ability, and in rare cases, delayed device failure.^[^ ^34^, ^35^, ^47^, ^48^, ^49^, ^50^, ^51^ ^]^ This clinical issue is anticipated to be addressed through sustained Dex release using a multicargo porous electrode drug‐loading coating design. At 28 days post‐CI in guinea pig cochlea, we assessed the degree of fibrosis in cochlea subjected to different treatments using immunofluorescence (**Figure** **7**). The results revealed that the cCI group (Figure 7a), the GelMA group (Figure 7c), and the GelMA + MXene group (Figure 7d) exhibited significant fibrotic tissue proliferation within the ST of the cochlea. In contrast, the degree of fibrosis was markedly reduced in the Dex group (Figure 7b) and the GelMA + MXene + Dex group (Figure 7e). To precisely analyze the differences among 5 groups, we calculated the ratio of fibrotic tissue area to the total area of the ST, as shown in Figure 7f–i. In the apical and upper middle turns of the cochlea, fibrosis was observed in the cCI and GelMA groups, while no significant fibrosis was detected in the Dex, GelMA + MXene, and GelMA + MXene + Dex groups. Statistical analysis revealed no significant differences in fibrosis levels among the 5 groups in these regions. In the lower middle turn, the cCI, GelMA, and GelMA + MXene groups exhibited mild fibrosis, whereas the Dex and GelMA + MXene + Dex groups showed no apparent fibrosis. However, no significant differences in fibrosis were observed among the 5 groups in this region. In contrast, in the basal turn, which is closest to the electrode implantation site, the cCI, GelMA, and GelMA + MXene groups displayed significant fibrosis, while the Dex and GelMA + MXene + Dex groups showed no detectable fibrosis, with a statistically significant reduction in fibrosis compared to the control groups. These findings suggest that the Dex and GelMA + MXene + Dex coatings are highly effective in suppressing fibrosis in the cochlear. Given that our animal experiments were conducted over a 28‐day period post‐CI, it is important to note that fibrosis is a progressive process that develops over time. Our results demonstrate that Dex exhibits promising efficacy in inhibiting fibrosis, indicating its potential as a therapeutic option for mitigating fibrosis‐related pathological changes following CI. Systematic comparison with previously reported studies highlights the superior performance of our multicargo coating in protecting SGNs, preserving residual hearing, and reducing fibrosis, offering valuable insights for enhancing cochlear implant electrode performance (shown in Table S1, Supporting Information). Further long‐term studies are warranted to validate these findings and explore the sustained benefits of this approach.

## 3 Conclusion

In summary, we proposed a novel porous electrode drug‐loading coating aimed to improve their compatibility within organisms. The process involved initial modification of the porous MA‐PDMS structure on the electrode surface, followed by filling the MA‐PDMS pores with GelMA hydrogel containing Dex and MXene. In vitro experiments confirmed the good biocompatibility and oxidation resistance of the porous electrode drug‐loading coating, while in vivo experiments demonstrated its protective effect on SGNs. In addition, the porous electrode drug‐loading coating could better preserve residual hearing, especially in the low‐frequency range, and effectively reduce fibrosis following CI. We hope that this novel porous electrode drug‐loading coating can provide a valuable reference for the application of the new cochlear electrode transplantation system.

## 4 Experimental Section

### Materials

Guinea pigs (200–230 g) and neonatal (P2‐P3) mice were purchased from Qinglongshan Animal Breeding Company (Nanjing, China). The cochlear electrode was purchased from MED‐EL Elektromedizinische Geräte GmbH (Austria). The Methacryloxypropyl)methylsiloxane‐dimethylsiloxane copolymer (MA‐PDMS) was purchased from Zhengzhou Gecko Scientific Inc (China). Dulbecco's modified eagle medium (DMEM) medium, DMEM/F12, 0.25wt.% trypsin, penicillin, phosphate buffered saline (PBS), Hank's Balanced Salt Solution (HBSS), Ethylenediaminetetraacetic acid (EDTA), collagenase, and fetal bovine serum (FBS) were purchased from GibcoBRL (Gaithersburg, MD, USA). Dexamethasone sodium phosphate, 2‐Hydroxy‐2‐Methyl‐1‐Phenyl Propanone (HMPP), Gelatin, methacrylic anhydride (MA), lithium fluoride (LiF), and N‐2‐hydroxyethylpiperazine‐N‐ethane‐sulphonicacid (HEPES) were purchased from Sigma–Aldrich Inc (Germany). NaOH, CaCl_2_, MgCl_2_, KCl, NaCl, HCl, glucose, span20, and liquid paraffin were purchased from Shanghai Aladdin Biochemical Technology Co.,Ltd. (China). B‐27, N2, and LIVE/DEAD Viability/Cytotoxicity Kit were purchased from Thermo Fisher Scientific Inc. (USA). Insulin‐like growth factor (IGF), epidermal growth factor (EGF) and fibroblast growth factor (FGF) were purchased from Stemcell Technologies Inc. (China). CCK‐8 and ROS detection kit were purchased from Beyotime Biotechnology (China).

### Synthesis of GelMA

To synthesize GelMA, 20 g of gelatin was first added into 200 mL of PBS solution and stirred continuously for 4 h. Subsequently, stirring of the mixture was continued in a water bath at 60 °C until the gelatin is fully dissolved. Next, 16 mL of MA was introduced into the system at a controlled rate of 0.25 mL per minute over the course of 2 h using a microsyringe pump. Once the addition was complete, the reaction was terminated by incorporating 800 mL of PBS. Following this step, the GelMA solution was subsequently dialyzed with deionized water at 37 °C for 7 days. The dialyzed solution was finally freeze‐dried to obtain the dried GelMA product.

### Synthesis of Ti_3_C_2_T_x_ MXene

The hydrothermal reactor kettle was cleaned using a NaOH solution followed by thorough rinsing with deionized water. Subsequently, 1 g of LiF, along with equal amounts of deionized water and 10 mL of HCl, was added to the dried hydrothermal reactor kettle and mixed using a magnetic stirrer. MAX material (1 g) was then gradually added to the stirred mixture. Afterward, the hydrothermal reaction kettle was securely wrapped with plastic film and positioned in a water bath at a constant temperature of 40 °C, with continuous magnetic stirring for at least 24 h. After the hydrothermal treatment was completed, the liquid was carefully transferred to a centrifuge tube and centrifuged at 3500 revolutions per minute (rpm) for 10 min. After removing the supernatant, the residue was re‐dispersed in deionized water and subjected to subsequent rounds of centrifugation. The centrifugation process was repeated incrementally, with the speed increasing by 500 rpm per cycle until a final centrifugation speed of 7500 rpm was reached. Ultimately, through multiple rounds of centrifugation and resuspension in deionized water, the Ti_3_C_2_T_x_ MXene solution was successfully obtained.

### Synthesis of Porous MA‐PDMS and Porous Electrode

Liquid paraffin was employed as the porogen for the preparation of porous MA‐PDMS. In a typical preparation process, an appropriate quantity of surfactant span20 was thoroughly mixed with the MA‐PDMS base. Liquid paraffin was then added to the above mixture several times, resulting in a final porogen volume fraction of 0.2–0.25. To ensure a homogeneous and stable emulsion, a suitable amount of n‐hexane was added during the addition of the porogen. Once a homogeneous emulsion was achieved, the photoinitiator HMPP (volume fraction of 0.01) was added and thoroughly mixed. Subsequently, the cochlear electrode was immersed in the mixture and cured under ultraviolet light. After that, the obtained electrode was immersed in anhydrous ethanol five times to remove the liquid paraffin for five times.

### Synthesis and Characterization of Porous Electrode Filled with GelMA‐MXene‐Dex Hydrogels

To prepare porous Electrode filled with GelMA‐MXene‐Dex hydrogels, porous electrodes with a length of 10 mm were employed, featuring a marked point at both 3 and 6 mm from the base. The diameter of the electrodes was tapered gradually, starting at 0.2 mm at the distal end and expanding to 0.3 mm at a distance of 6 mm from the tip. A 10% (w/v) GelMA hydrogel containing 400 µg mL^−1^ MXene was combined with Dex powder in varying mass ratios (95:5, 90:10, 80:20). The mixture was then dissolved in artificial perilymph at ambient temperature (37 °C). Subsequently, the electrode ≈10 mm in length was immersed in the hydrogel mixture for 10 s, and the GelMA‐MXene‐Dex hydrogel was filled into the MA‐PDMS holes of the porous electrode. The coated electrodes were left to air‐dry at room temperature for 24 h before further characterization.

### Swelling and Degradation Test

To evaluate the swelling properties of hydrogels, various concentrations of MXene (0, 25, 50, 100, 200, 400 µg mL^−1^) were introduced into GelMA hydrogel (10% w/v), followed by solidifying and immersing in PBS solutions for swelling testing. The lyophilized samples were weighed at specific time intervals. The swelling rate (WR) of the different hydrogel was calculated using the equation:

(1) $$WR=\left[\left(W_{a}-W_{0}\right)/W_{0}\right]\;\times 100\%$$

where W_0_ denotes the initial dry weight of the hydrogel and W_a_ represents the weight of the swollen sample after drying.

For assessing the degradation rate (DR) of GelMA‐MXene hydrogels, the hydrogels were immersed in a PBS solution containing 1 U /mL collagenase to creat an in vitro degradation environment. The lyophilized samples were then weighed at specific time points. The DR of the hydroges was calculated using the equation:

(2) $$DR=\left[\left(W_{0}-W_{b}\right)/W_{0}\right]\;\times 100\%$$

where W_0_ denotes the initial dry weight and W_b_ indicates the dry weight after degradation.

### Drug Loading and Release

The GelMA‐MXene‐Dex hydrogels were immersed in 3.8 mL of artificial perilymph composed of 1.3 mm CaCl_2_, 1.8 mm MgCl_2_, 5.4 mm KCl, 137 mm NaCl, 5 mm glucose, and 5 mm HEPES at 37 °C to allow Dex release. Artificial perilymph samples of 100 µL were taken at various time points ranging from 6 h to 40 days to monitor the levels of Dex. Following each measurement, an equal volume of fresh artificial perilymph were added to maintain the total volume of the solution. The Dex content and concentration in the artificial perilymph were measured at a wavelength of 242 nm using a UV spectrophotometer (Shimazu Instrument (Suzhou) Co., Ltd).

### Culture of HEI‐OC1 Cells

The HEI‐OC1 cells were maintained in a culture medium of DMEM supplemented with 10% FBS and 1% ampicillin, incubated under conventional conditions at 37 °C and 5% CO_2_. The culture medium was refreshed every 48 h.

### Culture of SGNs

Neonatal (P2‐P3) mice were anesthetized, sacrificed, and their temporal bones extracted under microscopic guidance in HBSS. The Organ of Corti was removed, leaving the modiolus, which was then placed in PBS. Subsequently, the modiolus underwent 8–10 min of 0.125% trypsin digestion at 37 °C, followed by gentle pipetting in culture medium to dislodge cells. The contained cells were then filtered for a single‐cell suspension using a 40 µm cell strainer. These cells were incubated in SGN_1_ medium at 37 °C with 5% CO_2_, and on the following day, the culture medium was switched to SGN_2_, which was changed every other day thereafter. The SGN_1_ medium was composed of DMEM/F12, 10% FBS, and 50 µg mL^−1^ ampicillin. The SGN_2_ medium was prepared by supplementing DMEM/F12 with B27, N2, 10 ng mL^−1^ FGF, 20 ng mL^−1^ EGF, and 50 ng mL^−1^ IGF.

### Biocompatibility Test

To assess the toxicity of GelMA‐MXene hydrogels on HEI‐OC1 cells and SGNs, CCK‐8 and Live/dead assays were conducted. For the CCK‐8 experiment, the diluted CCK‐8 solution was added to each well and incubated with cells for 2 h under standard conditions. The absorbance at 450 nm was then recorded using a microplate reader (Victor Nivo, PE). Moreover, the toxicity of different hydrogels was evaluated using the LIVE/DEAD Viability/Cytotoxicity Kit following the manufacturer's guidelines.

### ROS Measurement in Cells

ROS levels in HEI‐OC1 cells were determined using a ROS detection kit. Briefly, cells were incubated with 10 µm DCFH‐DA in serum‐free DMEM for in a confocal dish for 30 min at 37 °C. After washing with PBS three times, ROS were visualized under a ZEISS confocal microscope. ROS quantification was also achieved through flow cytometric asnalysis.

### Quantitative Real‐Time Polymerase Chain Reaction

Total RNA isolation from different cell populations was performed using commercial RNA extraction kits. The isolated RNA was then converted to cDNA through reverse transcription using the PrimeScript RT reagent Kit. RT‐qPCR analysis was conducted with LightCycler 480 SYBR Green I Master Mix, employing primer sequences provided in Table S2 (Supporting Information).

### Animal Surgery

The animal experiment was proved by Animal Experimental Ethics Committee of Southeast University (No. 20230301006). CIs were surgically implanted into one ear of each 5‐7‐week‐old guinea pig with normal hearing, following intraperitoneal injection of pentobarbital sodium (40 mg kg^−1^) for anesthesia. An ear posterior incision was made, and the bulla was accessed by retracting the muscle and skin, which was then carefully drilled open under microscopy. The round window membrane was then pierced using curved forceps, allowing a delicate 3‐mm insertion of the electrode into the ST. For the Dex group, 100 µL of 1 mg mL^−1^ Dex was infused into the ST after electrode placement. Guinea pigs in the cCI group received unmodified electrodes. For the GelMA, GelMA‐MXene, and GelMA‐MXene‐Dex groups, the porous electrodes filled with pure GelMA, GelMA‐MXene, and GelMA‐MXene‐Dex hydrogels were implanted into the cochleae of guinea pigs, respectively. To prevent perilymph leakage, the round window was sealed with a muscle fragment. Sutures were applied post‐surgery, and guinea pigs were returned to their cages.

### Auditory Brainstem Response

Hearing function was assessed using ABR tests. Anesthetized guinea pigs were positioned on a vibration‐dampened table in a soundproof and electromagnetically shielded room. Electrode placement involved three subcutaneous insertions: one at the top of the skull for signal pickup, another behind the tested ear as the reference, and the last one behind the contralateral ear serving as ground. Tone bursts (5 ms duration) of varying frequencies (4–32 kHz) and intensities were produced by SigGenRP software and presented at 10/s via a speaker positioned 15 cm from the ear. ABR signals were recorded, amplified, averaged, and stored using BioSigRZ software (Tucker‐Davis Technology) The non‐tested ear was plugged with a silicone earplug (Mack's) during recordings. Responses were averaged over 256 sweeps, and the ABR threshold was defined as the highest stimulus level at which no discernible response was observed. Upon recovery from anesthesia, the animals were returned to their cages.

### Immunofluorescence Staining and Confocal Imaging

At 28 days post‐surgery, the cochleae were fixed in 4% paraformaldehyde at 4 °C overnight. The samples underwent PBS washing before immersion in 0.5 m EDTA, with the solution being refreshed daily for a week. Following re‐washing with PBS, the cochleae were microscopically sectioned. To visualize HCs, immunofluorescence staining was perfomed using myosinVIIa and Phalloidin. The staining procedure involved fixation at room temperature for an hour, permeabilization for 15 min, and overnight incubation at 4 °C with diluted primary antibodies. The next day, the samples were washined with 0.1% Triton X‐100 in PBS (PBST), and were then incubated with secondary antibodies at room temperature for an hour. After washing with PBST, the samples were treated with an anti‐fading agent, sealed with nail polish, and preserved for subsequent observations and imaging. Another set of samples were embedded in optimal cutting temperature compound for cryosectioning, and 13µm‐thick sections were prepared utilizing a Leica cryostat. These sections underwent immunofluorescence staining following standard protocols for α‐SMA, Collagen I, Tuj‐1, and DAPI. Stained sections were observed and captured under a Zeiss 700 confocal microscope.

### Statistical Analysis

Image analysis was conducted using ImageJ software, and GraphPad Prism was utilized for data visualization and statistical analysis. The experimental were carried out in three repetitions or more and examined using Student's t‐test for pairwise comparisons and one‐way ANOVA accompanied by Dunnett's multiple comparison tests for analyses involving three or more groups. All results are presented as mean ± SD or mean ± standard error of the mean (SEM), as appropriate, with statistical significance set at *p* < 0.05.

## Conflict of Interest

The authors declare no conflict of interest.

## Author Contributions

L.R., Y.H., X.D., M.L., and T.S. contributed equally to this work. L.C., R.C., W.L., H.W., and L.L. conceived the idea and designed the experiment. L.R., H.Z., X.D., M.L., T.S., S.C., Y.Q., H.C., and P.F. carried out the experiments. L.R., Y.H., and H.W. analyzed data and wrote the paper.

## Supporting information



Supporting Information

## Data Availability

The data that support the findings of this study are available in the supplementary material of this article.
