# Supplementary material for: Multicargo Porous Cochlear Electrode Coating for Antifibrosis After Cochlear Implantation
Source: Adv Sci (Weinh). 2025 May 12;12(21):2412158. doi: 10.1002/advs.202412158 (PMC12140376; doi:10.1002/advs.202412158)
Supplement: Supplementary file 1 — Supporting Information [file ADVS-12-2412158-s001.docx]

**Supplementary**

**Multicargo Porous Cochlear Electrode Coating for Antifibrosis after Cochlear Implantation**

Lei Ren^1,2,#^, Yangnan Hu^1,^*^#^, Xiaoqiong Ding^1,#^, Menghui Liao^1,#^, Tian Shen^3,#^, Sixing Cao^1^, Hui Zhang^1^, Hong Cheng^1^,Yanru Qi^1^, Pan Feng^1^, Ling Lu^1,^*,Huan Wang^4,^*, Wenwen Liu^5,^*, Renjie Chai^1,2,6,7,8,^*, Lin Cheng^7,^*

1. State Key Laboratory of Bioelectronics, Department of Otolaryngology Head and Neck Surgery, Zhongda Hospital, School of Life Sciences and Technology, Advanced Institute for Life and Health, Jiangsu Province High Tech Key Laboratory for Bio-Medical Research, Southeast University, Nanjing 210096, China

2. Co-Innovation Center of Neuroregeneration, Nantong University, Nantong 226001, China

3. Department of Otolaryngology-Head & Neck Surgery, West China Hospital, Sichuan University, Chengdu 610041, China

4. The Eighth Affiliated Hospital, Sun Yat-Sen University, Shenzhen 518033, China

5. Department of Otolaryngology-Head and Neck Surgery, Shandong Provincial ENT Hospital, Shandong University, Jinan, 250022, China

6. Department of Neurology, Aerospace Center Hospital, School of Life Science, Beijing Institute of Technology, Beijing 100081, China

7. Department of Otolaryngology Head and Neck Surgery, Sichuan Provincial People's Hospital, University of Electronic Science and Technology of China, Chengdu, 610000，China

8. Southeast University Shenzhen Research Institute, Shenzhen 518063, China

Email:[101300306@seu.edu.cn;](mailto:101300306@seu.edu.cn;)[101013882@seu.edu.cn](mailto:101013882@seu.edu.cn); [wangh679@mail.sysu.edu.cn;](mailto:wangh679@mail.sysu.edu.cn;) [wenwenliu@email.sdu.edu.cn](mailto:wenwenliu@email.sdu.edu.cn); [renjiec@seu.edu.cn](mailto:renjiec@seu.edu.cn); chl2011@ustc.edu.cn.


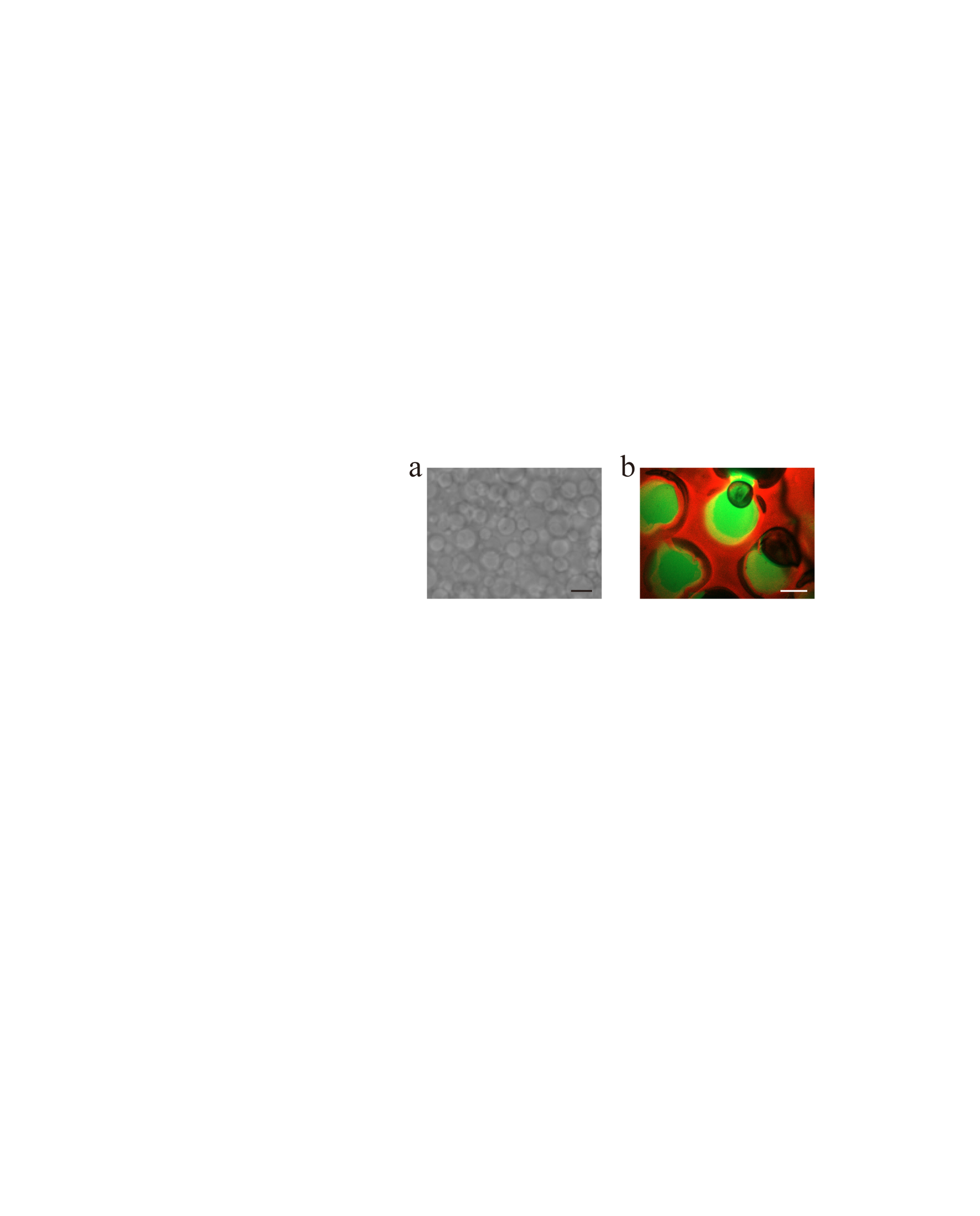


**Figure S1.** The visualized structure of the multicargo coating. a) The optical micrograph of porous MA-PDMS after hydrogel infiltration, with a scale bar of 50 μm. b) The confocal microscopy image of MA-PDMS (stained red) infiltrated with GelMA hydrogel (stained green), with a scale bar of 20 μm.


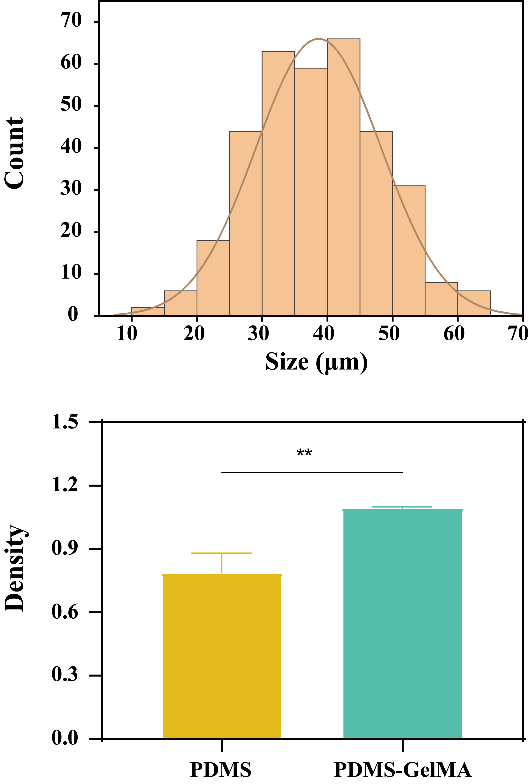


**Figure S2.** The pore size distribution analysis of the porous MA-PDMS.


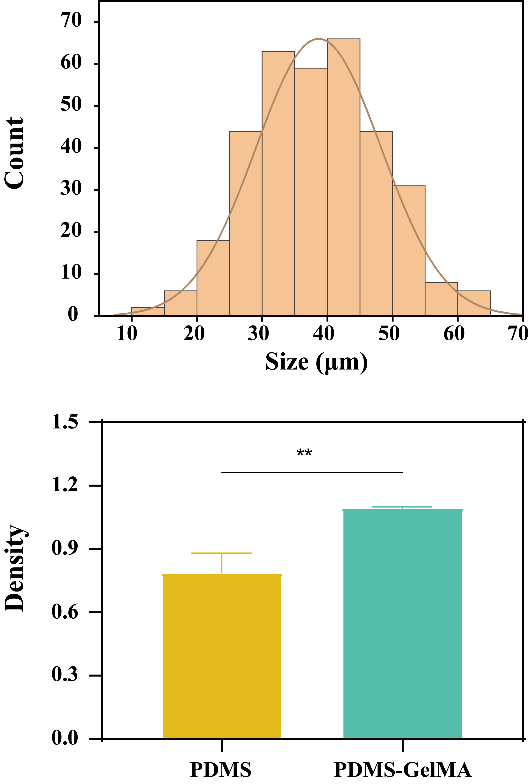


**Figure S3.** The density variation between porous MA-PDMS and porous MA-PDMS infused with GelMA hydrogel (n = 3). Data are presented as mean ± SD. p > 0.05; **p < 0.01, Student's t-test.


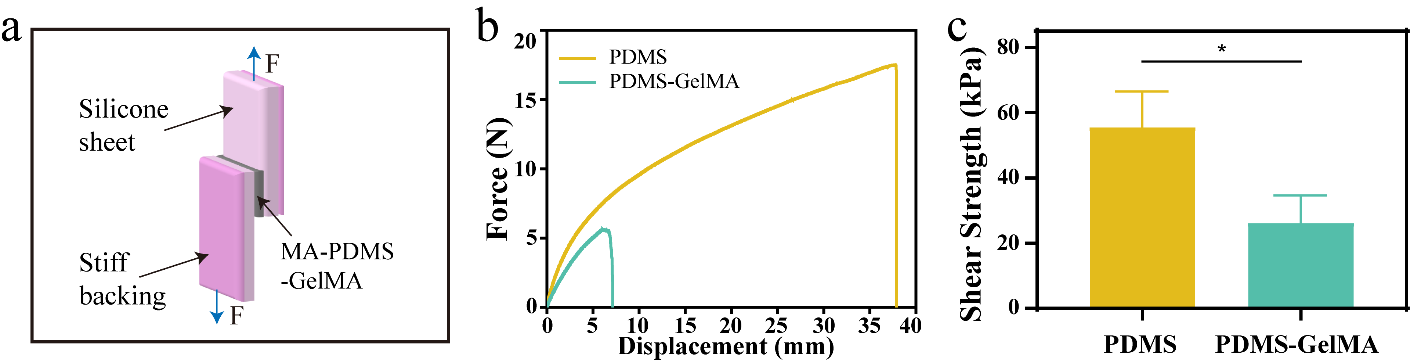


**Figure S4**. Measurement of shear strength of porous MA-PDMS and porous MA-PDMS infused with GelMA hydrogels. a) Schematic illustration the adhesion of two materials on silicone sheet for test. b) The force - displacement curves for two materials. c) The histogram of the shear strength for two materials (n = 3). Data are presented as mean ± SD. p > 0.05; *p < 0.05, Student's t-test.


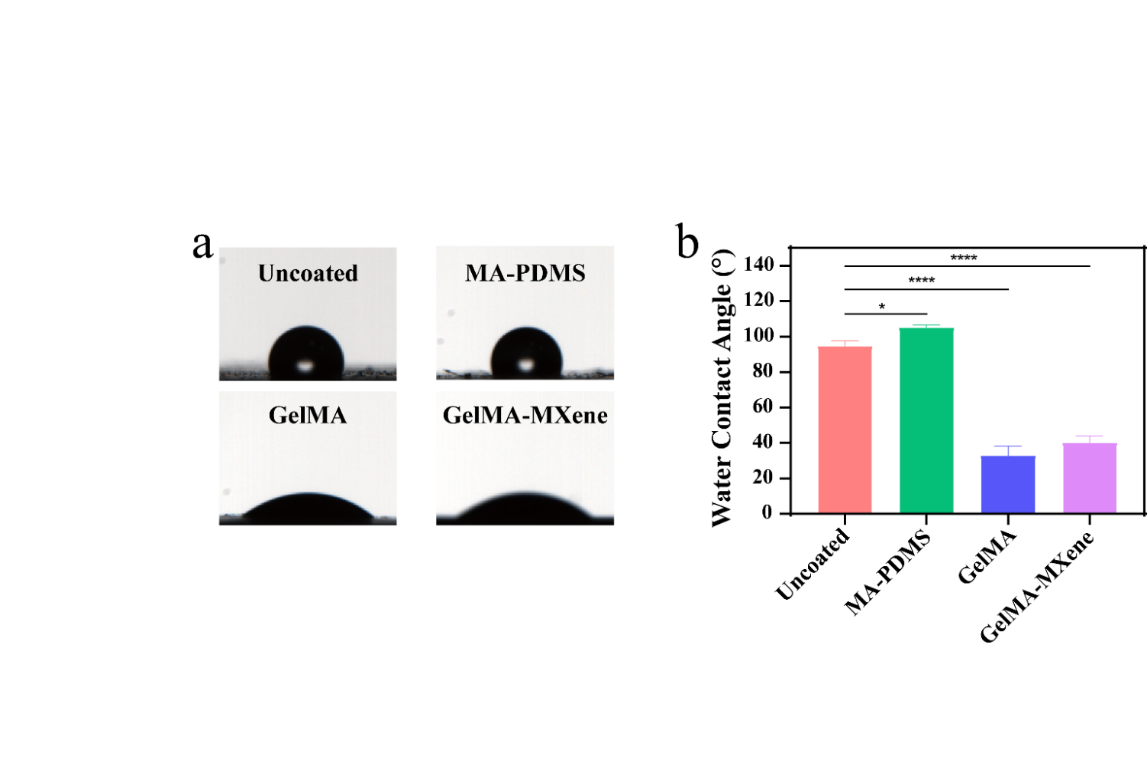


**Figure S5**. The multicargo coating exhibits super-hydrophilicity. a) Water contact angles for the uncoated group, porous MA-PDMS group, GelMA hydrogel-infused porous MA-PDMS group, and GelMA-MXene hydrogel-infused porous MA-PDMS group. b) Comparison of water contact angles among different groups (n = 3). Data are presented as mean ± SD. p > 0.05; *p < 0.05, ****p < 0.0001, one-way ANOVA.


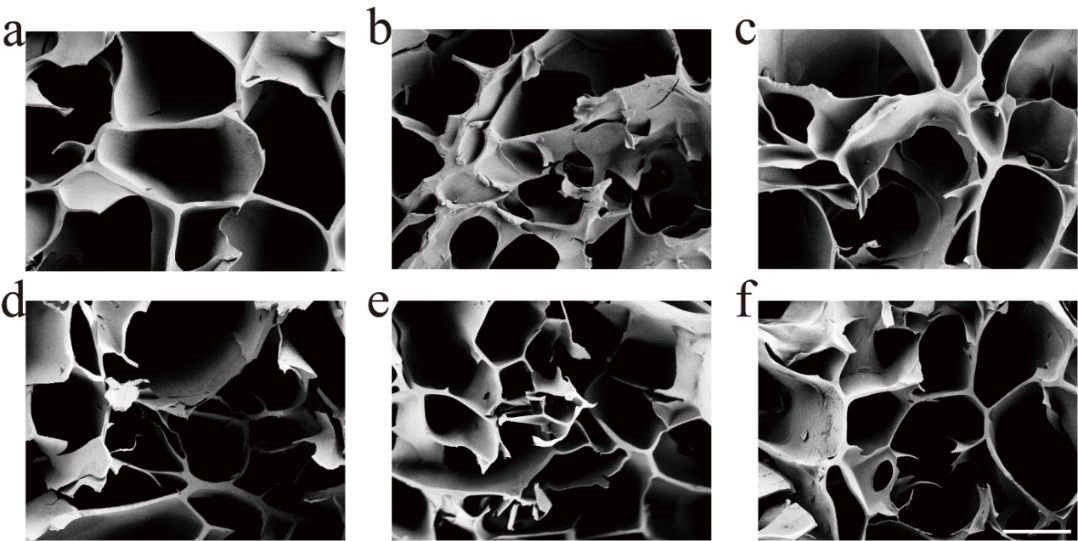


**Figure S6.** SEM images of the GelMA-MXene hydrogel; the MXene concentrations are 0, 25, 50, 100, 200, and 400 μg/mL from (a) to (f), respectively. The scale bar indicates 100 μm.


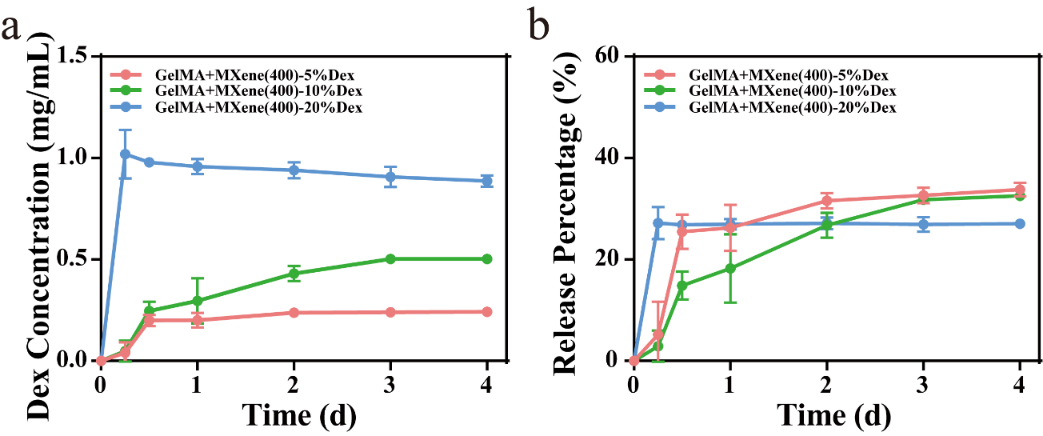


**Figure S7.** The release behavior of Dex from the GelMA-MXene-Dex hydrogels. (a) The real-time Dex concentration from the GelMA-MXene-Dex hydrogels for initial 4 days (n = 3). (b) The release percentage of Dex from the GelMA-MXene-Dex hydrogels for initial 4 days (n = 3). Data are presented as mean ± SD.


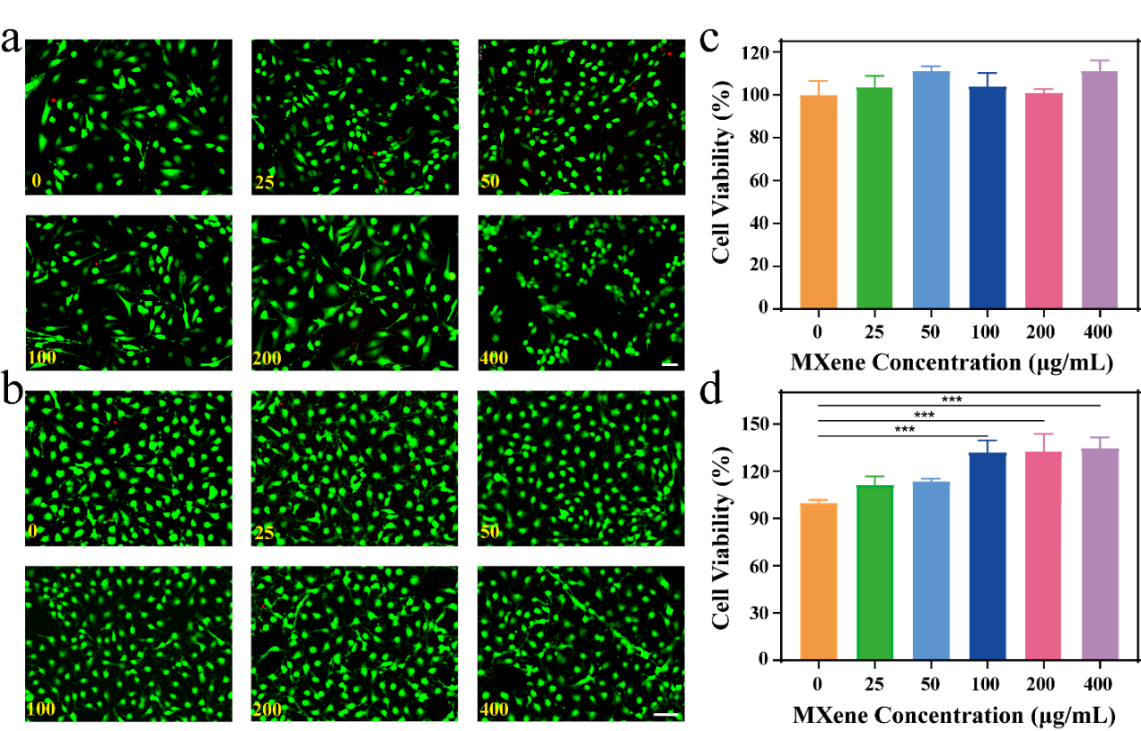


**Figure S8.** (a) Representative images of the HEI-OC1 cells cultured with GelMA-MXene hydrogels with different MXene concentrations for 48 hours. (green: live cells; red: dead cells). Scale bar: 50 μm. (b) Representative images of SGNs cultured with GelMA-MXene hydrogels with different MXene concentrations for 48 hours in vitro. (green: live cells; red: dead cells). Scale bar: 50 μm. (c, f) Cell viability of HEI-OC1 cells (c) and SGNs (d) from CCK-8 assay (n = 3). Data are presented as mean ± SD. p > 0.05; ***p < 0.001, one-way ANOVA.


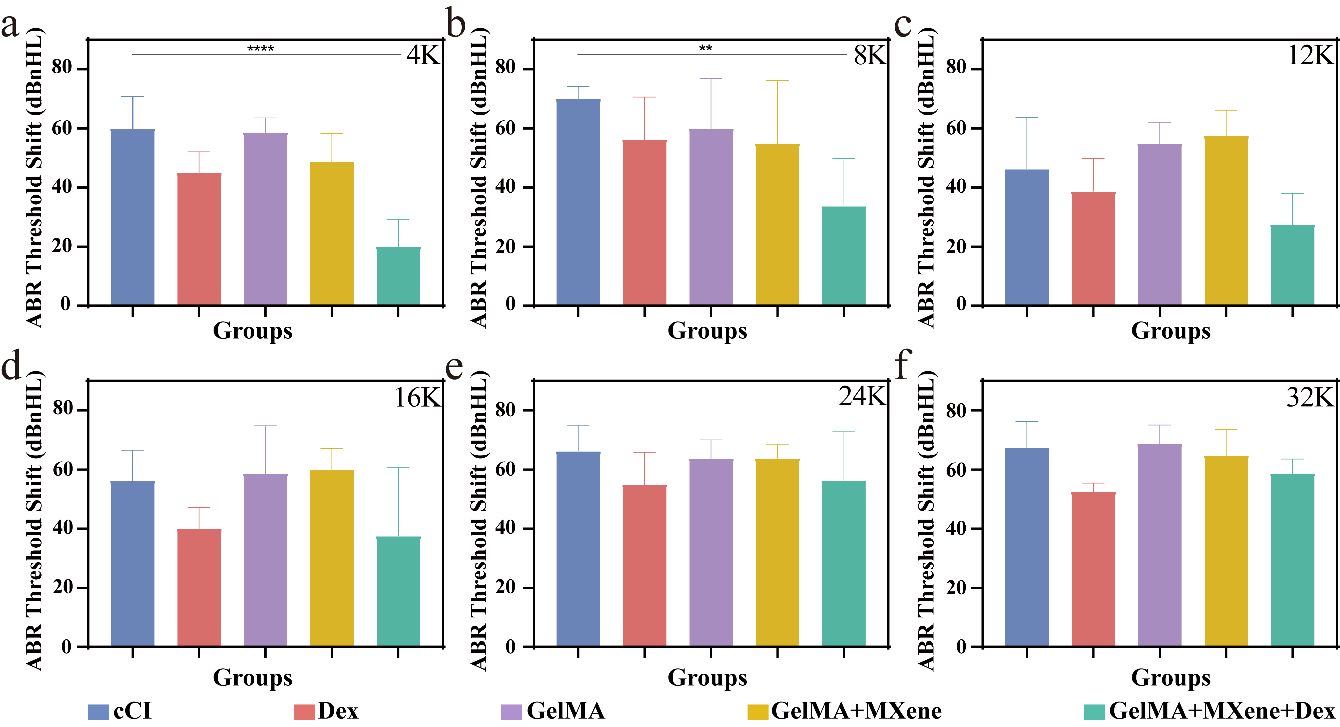
**Figure S9.** The hearing threshold shift of guinea pigs. The hearing threshold shift of 4K Hz

(a), 8K Hz (b), 12K Hz (c), 16K Hz (d), 24K Hz (e), and 32k Hz (f) 28 days after CI. (n = 4). The baseline hearing level of each group of guinea pigs was measured 7 days before CI. From left to right, groups are the cCI, the Dex, the GelMA, the GelMA+MXene, and the GelMA+MXene+Dex, respectively. Data are presented as mean ± SD. p > 0.05; **p < 0.01; ****p < 0.0001, one-way ANOVA.

Table S1. The comparison of our results with those reported in the existing literature.

| **Methods for Improving Cochlear Electrodes** | **Subjects** | **ABR** | **Hair cells / SGNs**  **/ Other Cells** | **Anti-fibrosis** | **References** |
| --- | --- | --- | --- | --- | --- |
| A porous MA-PDMS-coating filled with GelMA-MXene-Dex hydrogels on the surface of the cochlear electrode | Guinea pigs | 4kHz/8kHz threshold decreased in GelMA-MXene-Dex group (4 weeks after CI) | No significant loss of HCs for all groups; lower SGN loss in GelMA-MXene-Dex group from the apical to the basal turn. | Lower rate of fibrosis in GelMA-MXene-Dex group (4 weeks after CI) | - |
| Poly sulfobetaine methacrylate (PSB)-polydopamine (PDA) coating on cochlear electrode array | Sprague Dawley rat | 2–16 kHz threshold decreased in PBS-PDA group (4 weeks after CI) | - | Thinner fibrous capsule around implants and lower fibrosis rate of cochlear in PSB-PDA group (4 weeks after CI) | 25 |
| Silicone-based cochlear implant electrode arrays containing with micronized Dex | Guinea pigs | ABR and compound action potential thresholds with 10% and 1% Dex electrodes returned to near pre-CI levels, while 0.1% Dex showed only moderate improvement (90 days after CI) | The 1% and 10% Dex electrodes protected OHCs and nerve fibers from the basal to apical turns, while 0.1% Dex provided only partial protection (90 days after CI) | Lower fibrosis rate of cochlear in 1.0% or 0.1% Dex electrodes group (90 days after CI) | 26 |
| Intratympanic dexamethasone-loaded hydrogels combined with dexamethasone-eluting cochlear electrodes | Guinea pigs | No significant difference of ABR threshold between the groups. | No significant loss of HCs and SGNs between the groups | No significant differences of fibrosis rate between the groups. | 27 |
| Polylactic-co-glycolic acid (PLGA)-Dex coating on cochlear electrode array | Guinea pigs | 8kHz/16kHz/32kHz threshold decreased in PLGA-Dex group (4 weeks after CI) | - | Lower rate of fibrosis in PLGA-Dex group (4 weeks after CI) | 28 |
| The PDMS, with precipitated Dex, served as an implant base material and was surface-coated with star-shaped polyethylene glycol prepolymer (sPEG) | Guinea pigs | No significant differences between treatment groups (4 weeks after CI) | - | Lower connective tissue inside the scala tympani and on the CI surface in Dex-sPEG group and Dex group (4 weeks after CI) | 29 |
| The polycaprolactone (PCL) -Dex copolymer was coated on the tip of silicone dummy | Sprague Dawley rat | 2 kHz threshold decreased in PCL-Dex group (5 weeks after CI) | Lower HC loss from the apical to basal turns and lower SGN loss at the middle and basal turns in PCL-Dex group (5 weeks after CI) | Lower fibrosis rate of cochlear in PCL-Dex group (5 weeks after CI) | 30 |
| A silicone rubber rod coated with Dex-loaded polytrimethylene carbonate (PTMC) to simulate the electrode. | Wistar rats | - | - | Thinner fibrous capsule around implants in Dex-PTMC group (1 & 3 weeks after CI) | 31 |
| A silicone rubber rod coated with Dex-loaded PCL to simulate the electrode. | Sprague Dawley rat | - | - | Weaker neutrophil infiltration in PCL-Dex group (1 & 3 weeks after CI) | 32 |
| Electrode array coated with AC102 | Female Mongolian gerbils | 2.5-8 kHz CAP threshold decreased in AC102-EI group (4 weeks after CI) | Lower OHC and IHC loss in basal turn for AC102-EI group; lower Type-II auditory nerve fibers loss at 8.0 kHz and 4.0 kHz for AC102-EI group  (4 weeks after CI) | - | 33 |
| Laminin-coated silicone electrode array | Sprague Dawley rat | 8/16/32 kHz threshold decreased in laminin-coated group (4 weeks after CI) | Lower SGN loss in laminin-coated group (4 weeks after CI) | - | 34 |
| Poly (3, 4-ethylenedioxythiophene) /arginine-glycine-aspartic acid -alginate hydrogel/BDNF-coated cochlear electrode. | Guinea pigs | - | No significant difference in SGN survival between the coated and uncoated groups. | - | 35 |
| Cochlear electrode analogs, PDMS filaments loaded with Dex and coated with hyaluronic acid | L929 mouse fibroblast cells | - | Excellent anti-fibroblast cell proliferation effects | - | 36 |
| The cross-linked hydrogel (Hyaluronic acid crosslinked with 1, 4-butanediol diglycidyl ether) coated the cochlear electrode. | SGN explants | - | No significant differences of morphology of SGN neurite bundles between the control and hydrogel groups | - | 37 |
| A metallic electrode surface deposite phosphate nanoparticles loaded with nucleic acids. | SGN explants | - | Dispersed calcium phosphate nanoparticles could transfected SGNs with eGFP or BDNF plasmids, but failed to achieve transfection from coated surfaces | - | 38 |

Table S2: The primer sequences information

| **Primer** | **Sequence (5'to3')** |
| --- | --- |
| β-actin Forward sequence | CATTGCTGACAGGATGCAGAAGG |
| β-actin Reverse sequence | TGCTGGAAGGTGGACAGTGAGG |
| GPX4 Forward sequence | CCTCTGCTGCAAGAGCCTCCC |
| GPX4 Reverse sequence | CTTATCCAGGCAGACCATGTGC |
| SOD1 Forward sequence | GGTGAACCAGTTGTGTTGTCAGG |
| SOD1 Reverse sequence | ATGAGGTCCTGCACTGGTACAG |
| HO-1 Forward sequence | CACTCTGGAGATGACACCTGAG |
| HO-1 Reverse sequence | GTGTTCCTCTGTCAGCATCACC |
